# Development and validation of a survey to measure features of clinical networks

**DOI:** 10.1186/s12913-016-1800-0

**Published:** 2016-09-30

**Authors:** Bernadette Bea Brown, Mary Haines, Sandy Middleton, Christine Paul, Catherine D’Este, Emily Klineberg, Elizabeth Elliott, on behalf of the Clinical Networks Research Group

**Affiliations:** 1 Sax Institute, PO Box K617, Haymarket, NSW 1240 Australia; 2 School of Public Health, University of Sydney, Sydney, NSW 2006 Australia; 3 Nursing Research Institute, St. Vincent’s Health Australia (Sydney) and Australian Catholic University, Executive Suite, Level 5, St Vincent’s Hospital, deLacy Building, Victoria Street, Darlinghurst, NSW 2010 Australia; 4 School of Medicine and Public Health, Priority Research Centre for Health Behaviour & Hunter Medical Research Institute, University of Newcastle, University Drive, Callaghan, NSW 2308 Australia; 5 National Centre for Epidemiology & Population Health, Research School of Population Health, ANU College of Medicine, Biology & Environment, Australian National University, Building 62, Cnr of Eggleston and Mills Roads, Canberra, ACT 2601 Australia; 6 New South Wales Health, 73 Miller Street, North Sydney, NSW 2060 Australia

**Keywords:** Clinical networks, Survey, Reliability, Validity, Leadership, Engagement, Strategic management, External support, Organisational change

## Abstract

**Background:**

Networks of clinical experts are increasingly being implemented as a strategy to improve health care processes and outcomes and achieve change in the health system. Few are ever formally evaluated and, when this is done, not all networks are equally successful in their efforts. There is a need to formatively assess the strategic and operational management and leadership of networks to identify where functioning could be improved to maximise impact. This paper outlines the development and psychometric evaluation of an Internet survey to measure features of clinical networks and provides descriptive results from a sample of members of 19 diverse clinical networks responsible for evidence-based quality improvement across a large geographical region.

**Methods:**

Instrument development was based on: a review of published and grey literature; a qualitative study of clinical network members; a program logic framework; and consultation with stakeholders. The resulting domain structure was validated for a sample of 592 clinical network members using confirmatory factor analysis. Scale reliability was assessed using Cronbach’s alpha. A summary score was calculated for each domain and aggregate level means and ranges are reported.

**Results:**

The instrument was shown to have good construct validity across seven domains as demonstrated by a high level of internal consistency, and all Cronbach’s α coefficients were equal to or above 0.75. In the survey sample of network members there was strong reported commitment and belief in network-led quality improvement initiatives, which were perceived to have improved quality of care (72.8 %) and patient outcomes (63.2 %). Network managers were perceived to be effective leaders and clinical co-chairs were perceived as champions for change. Perceived external support had the lowest summary score across the seven domains.

**Conclusions:**

This survey, which has good construct validity and internal reliability, provides a valid instrument to use in future research related to clinical networks. The survey will be of use to health service managers to identify strengths and areas where networks can be improved to increase effectiveness and impact on quality of care and patient outcomes. Equally, the survey could be adapted for use in the assessment of other types of networks.

**Electronic supplementary material:**

The online version of this article (doi:10.1186/s12913-016-1800-0) contains supplementary material, which is available to authorized users.

## Background

Clinical networks are burgeoning internationally and have been established in the United States, United Kingdom and other parts of Europe, Australia and Canada [1–10]. These networks aim to engage clinicians in the implementation of quality improvement initiatives [2, 3, 5, 8, 11] and there are data suggestive of networks being effective in improving quality of patient care [2, 5, 7, 12]. While there are many different models of clinical network from fully integrated service delivery systems, such as Kaiser Permanente or the Veterans Health Administration in the US, to informal communities of practice, all share the aim of increasing the uptake of evidence based practice and improving quality of care and patient outcomes. In the current context, we define clinical networks as voluntary clinician groupings that aim to improve clinical care and service delivery using a collegial approach to identify and implement a range of strategies across institutional and professional boundaries [13].

The effectiveness of clinical networks is often not formally evaluated. Published studies typically focus on one clinical area and provide anecdotal, experiential commentary using a mixed methods approach (e.g. document review, interviews, observation) [14–17]. The psychometric properties of measures have rarely been explored or tested, resulting in a lack of standard or validated methodology.

A recent systematic review of measurement instruments developed for use in implementation science (specifically to measure self-report research utilisation) found a large majority of instruments demonstrated weak psychometric properties [18]. Basic psychometric properties of reliability (e.g. internal reliability) and validity (e.g. construct validity) should generally be evaluated if a measure is to be implemented for research [19].

Given the rapid development and investment in clinical networks internationally [20, 21] there is a need to develop valid instruments to assess intrinsic and extrinsic features related to their performance. The aim of this paper is to outline the development, validation and descriptive results of an Internet survey designed to assess the effectiveness of clinical networks in order to guide future strategic and operational management and leadership in the wider context in which they operate. The survey was used in an Australian study involving 19 clinical networks of the Agency for Clinical Innovation [13]. The survey was developed by building on the limited existing measures relating to clinical networks, the wider organisational literature, and findings of a qualitative pre-study [22]. This paper addresses the following:

1. Development of the survey instrument
2. Psychometric assessment of the survey instrument (construct validity and scale reliability)
3. Descriptive survey results from a sample of network members^1^

## Methods

### Context

In New South Wales (NSW), Australia, The Agency for Clinical Innovation (hereafter called the Agency) has established a coordinated program of over 30 managed clinical networks, institutes and taskforces. The networks are formed around specialty health service areas and serve a population of 7.5 million people [23]. These state-funded clinical networks have a system-wide focus where members identify and advocate for models of service delivery (e.g. outreach services, new equipment, using technology to improve diagnosis) and quality improvement initiatives (e.g. guideline development and dissemination, training and education for health professionals) [24–27]. The networks have a consistent organisational structure; medical, nursing and allied health clinicians act in a voluntary capacity as co-chairs while salaried network managers provide operational level support. The Agency executive works across all networks and provides high-level strategic and technical assistance. The NSW clinical networks have similarities to clinical networks that operate in other countries and other jurisdictions within Australia in that they are virtual entities designed to drive increases in standards of patient care through integration of services and collaboration.

### Sample

The survey was administered as part of a broader study conducted in partnership with the Agency, investigating the factors associated with successful clinical networks, reported elsewhere [13]. The survey was used to collect data from members of 19 clinical networks established by the Agency that covered the clinical areas of: aged care, bone marrow transplantation, brain injury, cardiac, endocrine, gastroenterology, gynaecological oncology, home enteral nutrition, neurosurgery, nuclear medicine, ophthalmology, radiology, renal, respiratory medicine, severe burn injury, spinal cord injury, stroke, transition care and urology.

### Data sources for instrument development – determining domains and question development

In developing the instrument, it was deemed important that it be suitable for timely completion within the context of members’ busy, resource stretched workplace. First we determined the domains of the survey and then developed questions to measure the domains.

1. ***Domain data sources:*** Domains to be measured by the instrument were derived over an 18-month period, mindful of their utility to inform future implementation research, from a number of sources of information as follows:

(i) ***A review of published literature*** on models of effective healthcare organisations and quality improvement. While there are many models that identify components of effective healthcare organisations, with many commonalities across models, Bate’s theory of change in healthcare [28] most closely corresponded to the context of the broader study for which the instrument was developed, and posits that there are three types of factors that combine to exert influence on the successful implementation of organisational change, namely:

- *Internal network organisational components such as the strength and quality of clinical leadership; the quality of internal management; and engagement of clinicians*

The effectiveness of all types of networks is influenced by their organisation, specifically: strong clinical leadership and engagement of clinicians; [17, 29, 30] and efficient internal management [14, 31]. Poor leadership has been found to explain slow or partial or failed quality improvement in a number of studies [17, 30, 32]. In the context of networks there is a need for a shift from bureaucratic, vertical, role based models of management towards a more flexible cross boundary, influence-based leadership style. Leaders are responsible for creating a vision of where the network is going and implementing initiatives to achieve that vision [33]. To be effective, leaders must be role models who are motivational, [34] engage with the external environment and build collaborative relationships [35]. Therefore, while some aspects of leadership may be measured objectively, in order to gain a complete assessment it is essential to assess these factors via the perceptions of those with direct experience of the individual or team of interest.

- *Well-designed quality improvement initiatives*

Networks have a common need for well-designed quality improvement programs and support from the context in which they operate. Ideally, programs for networks would be based on analysis of the problem, address a specific targeted structural or behavioural change, have an evidence-based implementation plan and monitor impact [36, 37].

- *A high level of external support from hospital management*

Also important is a high level of external support from hospital management and wider health service regulatory bodies. Clinical networks operate within a complex political, cultural and organisational context [15] and complexity due to the involvement of multiple levels of government has been cited as a barrier to network success [38]. Research into both effective healthcare organisations and quality improvement programs stresses the importance of external support in bringing about change. In a review of existing literature on the determinants of the effectiveness of public networks, including clinical networks, Turrini and colleagues [39] identified cohesion in the local community, and local community support and participation as critical factors in the success of networks.

(ii)  
***A qualitative study*** of 27 key informants from the Agency to explore views about important outcomes and indicators of success of clinical networks as well as factors associated with their success. This study identified five key conditions important for the establishment of successful clinical networks: building relationships; effective leadership; strategic evidence-based work plans; adequate resources; and the ability to implement and evaluate network initiatives [22].
(iii)  
***A program logic framework*** that underpins the clinical network model of the Agency to illustrate the ways in which investment in and actions of the networks are anticipated to improve healthcare and health outcomes, at both the local clinician and health system level and system-wide [13].
(iv)  
**Consultation with Agency stakeholders** to determine if there were any other factors they regarded as important.

These sources all highlighted the importance of organisational and program factors and external support, which were the final domains measured by our Internet survey.

2. ***Question development:*** Questions developed to measure domains were derived, and in some cases adapted from, a selection of existing instruments [14, 28, 33, 40–42] where appropriate or designed by the research project investigator group based on themes arising from the qualitative study [22] and Agency stakeholder consultation. A summary of the source of each question can be found in Table 1.

**Table 1:** Summary of domains, their definitions and indicators

Domains included in final survey	Definitions	Indicators/questions
Perceived engagement of multidisciplinary clinicians	Engaging clinicians in networks is a fundamental part of their existence. Engagement is defined in terms of: ➢ Interdisciplinary collaboration ➢ Collaboration with consumers ➢ Engagement with rural/remote sector	➢ Number of hours devoted to network initiatives in last 6 months (estimate of time)^a^ ➢ Perceived commitment to network and belief in the work it undertakes [22] ➢ Perceived ability to contribute to driving the network agenda [22] ➢ Perceived contribution of views and ideas to network initiatives [22]
Perceived leadership of: 1. Network Manager 2. Network Co-chairs 3. Agency Executive	Strength and quality of the sub-categories of transformational and/or transactional leadership of the network, including network managers, co-chairs, and the Agency executive.	Transformational leadership: ➢ Vision and facilitation [22, 28] ➢ Motivation and role model [22, 28] ➢ Building collaborative relationships and engaging with the external environment [22, 33] Transactional leadership: ➢ Clearly defined goals and achievable work plans ➢ Ability to implement change [42]
Perceived strategic and operational management of a network	The efficiency of the internal management of the networks across the following dimensions: ➢ Composition of the network executive committee ➢ Open and facilitative approach to management ➢ Clearly stated written governance and decision-making processes (i.e. a strategic plan) Effective internal and external communication and organisational processes.	➢ Perceived multidisciplinary representation [22] ➢ Perceived dominance of individuals [14] ➢ Network environment perceived as supportive [14] ➢ Perceived effectiveness of information sharing across the network [40] ➢ Perceived effectiveness of communication with people outside the network [22] ➢ Perceived organisational ability of the Network Manager [22]
Perceived external support	The alignment of network agendas and facilitative relationships with stakeholders external to the network, including NSW Health, Area Health Services (AHS), and hospital management and staff.	➢ Support from hospital management [22] ➢ Awareness of and support for network ideas and initiatives by AHS Managers [22] ➢ Willingness of hospital clinicians to implement changes recommended by network^a^ ➢ Awareness of network recommendations by NSW Health [22] ➢ Network work plans and agendas aligned with state government strategic plans^a^
Network perceived as valuable	Perceptions about the worth of the Agency clinical networks, including the belief that the networks have the scope to make a contribution to health service provision in NSW.	➢ Perception that the network has improved quality of care [22] ➢ Perception that the network has improved patient outcomes [22] ➢ Perception that the network has led to system improvements [22] ➢ Likelihood of recommending colleagues join the clinical network^a^ ➢ Perception that network involvement is of benefit professionally [41]

^a^Question developed by the investigator team

### Selection of domains and their measurement

Following synthesis of data sources, the research project investigator group comprised of health service, clinical and statistical experts selected seven domains that measured the organisational, program and external support features of networks that were consistently identified in the international published literature, and through local qualitative interviews, and stakeholder consultation, and hypothesised to be key explanatory factors along the causal pathway for outcomes of effective networks [13]. These were: perceived engagement of clinicians; perceived leadership of the network manager; perceived leadership of network co-chairs; perceived leadership of the Agency executive; perceived strategic and operational management of the network; perceived external support; and perceived value of the clinical network. Definitions of these domains are provided in Table 1. The survey questions had a five-point Likert response scale (‘strongly agree’ to ‘strongly disagree’ with an additional ‘don’t know’ option) where participants were asked to select the response that best reflected their opinion for each question. There were between 2 and 8 questions per domain.

### Instrument piloting

The instrument was formatted as an Internet survey programmed by The Webmart Network using their PUBLICeye™ platform. The survey was piloted in October 2011 with 163 members of a clinical network in operation in NSW comprised of a variety of occupational groups (doctors, nurses, and allied health professionals). This network was not eligible for inclusion in the broader study for which the survey was developed due to its more recent establishment (in 2010). Members, identified through the Agency’s records, were invited to participate via email with a link to the online survey. The survey was pilot tested for feasibility, acceptability and comprehension, and participants were asked to provide comment on the clarity of the questions and whether the survey wholly captured their views. The survey was refined with minor amendments based on the results of the pilot-test and feedback from respondents.

### Instrument implementation and testing

All members of the 19 clinical networks between 2006 and 2008 with a valid email address were emailed an invitation to participate in the survey in November 2011. A number of strategies to optimise response rate were used including personalised, email invitations endorsed by the incumbent Agency Chief Executive Officer followed by two subsequent email reminders. Recall aids (anchors, prompts and cues, and the use of multiple data sources to improve accuracy) were used within the survey to minimise potential recall and social desirability biases [43, 44]. A copy of the survey is provided in Additional file 1.

### Statistical methods

SAS 9.1 (SAS Institute Inc, Cary, NC, USA) and Stata 11 software (StataCorp, College Station, TX, USA) were used for analysis. A factor analysis was undertaken on the survey data questions to assess construct validity. As the aim of the factor analysis was to investigate the hypothesised structure of the instrument and the validity and reliability of each of the proposed domains, a separate confirmatory factor analysis was undertaken for each hypothesised domain, using the principle axis factoring (PAF) method with promax (oblique) rotation. This was considered to be more appropriate than undertaking an exploratory analysis of all questions, as it would provide information about the structure and factors as designed, rather than investigate alternative factor structures. Questions were considered for exclusion from a factor if they had a factor loading less than 0.4 with individual items reviewed for interpretability and logic prior to exclusion [45]. Cronbach’s alpha was obtained for each domain as a measure of internal consistency. Inter-question covariance >0.3 indicates a significant correlation between questions on a domain. Scale reliability coefficients were classified as: 0.7-0.8 - acceptable; 0.8-0.9 - good; 0.9-1.0 - excellent [46]. Likert scale response categories were collated for descriptive analyses, such that percentages of respondents who agreed/strongly agreed with items are reported as a single ‘agree’ category and disagreed/strongly disagreed are reported as ‘disagree’. For each domain, a total score was obtained for each individual by summing the values for all non-missing questions and dividing by the total number of questions completed; scores were only obtained if at least 50 % of the questions in the domain were completed. Aggregate means and ranges for summary scores are reported across the seven domains.

## Results

### Response rates and sample characteristics

Three thousand two hundred thirty-four members of 19 clinical networks with a valid email address were invited to participate in the survey. The survey response rate was 18 % (*n* = 592), which is less than the average response rate for online surveys reported at 33 % [47]. A summary of the demographic characteristics of respondents is presented in Table 2.

**Table 2:** Characteristics of study sample (*n* = 592)

Demographic characteristics		*n* (%)
Gender	Male	116 (32.0)
	Female	247 (68.0)
	Missing	229
Professional discipline	Medical Officer	91 (23.3)
	Nurse	150 (38.4)
	Consumer	13 (3.3)
	Allied Health	85 (21.7)
	Executive manager - non-health professional	6 (1.5)
	Researcher/academic	19 (4.8)
	Other	27 (6.9)
	Missing	201
Years involved in network	1	42 (8.2)
	2	60 (11.7)
	3	74 (14.5)
	4	55 (10.8)
	5+	280 (54.8)
	Missing	81
Role in network	Chair	12 (3.6)
	Executive Committee Member	24 (7.3)
	Executive & Steering Committee Member	17 (5.2)
	Expert Advisor	5 (1.5)
	Working group member	109 (33.1)
	Participant	162 (49.2)
	Missing	75

### Construct validity

In general the factor structure was consistent with the hypothesised domains. For the perceived engagement domain, two of the seven questions did not load well (factor loading <0.4) and these questions were excluded from calculation of the factor score for further analyses. The range of loadings for each domain, along with the means (and standard deviations) is shown in Table 3. Approximately two thirds (67 %) of the total variance was explained by the final factor solution.

**Table 3:** Outcomes of factor analysis for the seven hypothesised domains

Domain	No. of questions	No. of responses	Mean raw response	Standard deviation	Factor loadings (absolute value range)
Perceived engagement	5	445	3.3	0.68	0.651 – 0.827
Perceived leadership of network manager	7	314	3.9	0.78	0.392 – 0.922
Perceived leadership of network co-chairs	8	261	3.7	0.68	0.611 – 0.873
Perceived leadership of Agency Executive	2	317	3.8	0.80	0.958 – 0.958
Perceived strategic and operational management of a network	6	342	3.8	0.70	0.660 – 0.868
Perceived external support	7	228	3.3	0.60	0.503 – 0.802
Network perceived as valuable	5	340	3.8	0.78	0.684 – 0.902

Handling of missing data and summary scores: For those subjects with more than 50 % of the questions answered, a prorated individual total was calculated using the formula Prorated total = (Sum of question scores) x (n of questions in the subscale)/(n of questions answered). Those subjects with 50 % or more of the questions not answered were given a missing value for the scale. Domain scores were then calculated as the sum of the scores for each question within the factor and the network average score used in further analyses in the broader study

### Internal reliability estimations

Table 4 lists the Cronbach alpha coefficients for each of the seven domains within the instrument. Cronbach’s alpha ranged from 0.75 to 0.92 indicating that all of the seven survey domains exceeded the acceptable standard (>0.70), with five of those domains achieving high internal consistency [48].

**Table 4:** Survey internal reliability estimations

Domains	Number of questions	Average inter-question covariance	Scale reliability coefficient (Cronbach’s alpha)	Reliability
Perceived engagement	5	0.51	0.75	Acceptable
Perceived leadership of network manager	7	0.55	0.91	Excellent
Perceived leadership of network co-chairs	8	0.50	0.89	Good
Perceived leadership of Agency Executive	2	0.59	0.92	Excellent
Perceived strategic and operational management of a network	6	0.43	0.87	Good
Perceived external support	7	0.30	0.79	Acceptable
Network perceived as valuable	5	0.54	0.87	Good

### Descriptive results for the survey sample

Table 5 provides full details of mean summary scores and ranges across measured domains. Descriptive results for the survey sample are detailed in Additional file 2.

**Table 5:** Aggregate mean summary scores across domains

Domain	Mean summary score	Standard error of mean	Minimum-maximum	Maximum possible score
Perceived engagement	17.7	0.26	16.24 – 20.64	27
Perceived leadership of network manager	27.6	0.47	23.46 – 31.92	35
Perceived leadership of network co-chairs	29.6	0.44	25.33 – 32.52	40
Perceived leadership of Agency Executive	7.5	0.12	6.33 – 8.25	10
Perceived strategic and operational management of the network	22.9	0.23	21.22 – 24.85	30
Perceived external support	23.0	0.30	20.60 – 25.97	35
Network perceived as valuable	18.9	0.27	17.32 – 21.70	25

One third of survey respondents reported that they spent less than one hour per week devoted to network activities (33 %); one quarter (25 %) spent between one and five hours per week; 20 % between five and 10 h per week; 11 % between 10 – 20 h per week; and 11 % more than 20 h per week. The mean summary score for *perceived engagement* across networks was 17.7 out of a possible 27 (65.5 %). There was strong reported commitment to the network (73.5 %) and belief in the work that the network undertakes (86.7 %). However, there was less agreement that respondents’ views and ideas had contributed to network activities (55 %) or that they had been able to help drive the network agenda (30 %).

*Perceived leadership of network manager* had the highest mean summary score across the seven measured domains at 27.6 out of a maximum 35 (78.9 %), suggesting that, on the whole, network managers were considered to have an evidence-based vision (71 %), were able to engage fellow professionals about service and quality improvement (73.5 %) and bring others together to facilitate service and quality improvement (75.9 %). Network managers were perceived to have built strong positive relationships with clinicians (71.4 %) but were perceived by fewer respondents to have done so as effectively with consumers (49.1 %) or hospital management (38.9 %). Ratings of the *leadership of the network co-chairs* (29.6 out of 40; 74 %) were similar to those for network managers. Co-chairs were considered to be champions for change (63.8 %) and to have built strong, positive relationships with other clinicians (61.6 %) but less so with consumers (39.7 %) and hospital management (40.4 %). There was variability in perceptions of co-chairs’ abilities to mobilise fellow professionals about service and quality improvement (47.8 %), collaborate with external parties to support network operations (42.1 %) or work cooperatively with senior health department leadership to make appropriate changes (51.7 %). The summary score for *leadership of the Agency Executive* was 7.5 out of 10 (75 %). Just over half of respondents agreed that there was strong leadership and clear strategic direction (53.8 %) and that the Executive worked cooperatively with leaders in the wider health system to make appropriate changes (55.3 %). More than 40 % of respondents, however, selected a “neutral” or “don’t know” response for the two items within this domain.

*Perceived strategic and operational management of a network* had a mean summary score of 22.9 out of a possible 30 (76.4 %). The majority of respondents were satisfied with the level of multidisciplinary representation (81.8 %), the level of information sharing across the network (75.1 %) and to a lesser extent communication with people outside the network (55.8 %).

*Perceived external support* had the lowest summary score (23 out of 35; 65.7 %). Just over half agreed that network agendas were aligned with state government strategic plans (52.3 %). Fewer network members felt that hospital management (28.6 %), clinicians working in hospitals (50.3 %) and local area health service managers (15.9 %) were willing to implement network recommended changes despite more than a third reporting that area health service managers (34.4 %) and state government health decision makers (35.5 %) were aware of these recommendations.

Overall, the *networks* were *perceived as valuable* (18.9 out of 25; 75.6 %) and were considered by members to have improved quality of care (72.8 %) and, to a slightly lesser extent, patient outcomes (63.2 %). More than 70 % of respondents would recommend joining the network to a colleague.

## Discussion

Prior to the development of this network survey, to the best of our knowledge, there were no psychometrically validated surveys designed to measure the organisational, program and external support features of clinical networks. This paper describes the development and assessment of construct validity and internal reliability of a survey instrument, and provides descriptive results from a formative assessment of nearly 600 members of 19 diverse clinical networks across the seven measured domains. The survey was developed as an instrument to measure factors associated with successful clinical networks in an Australian study [13]. It provides researchers and managers of clinical networks with a psychometrically valid and reliable tool that can be used to assess key features of successful clinical networks and to identify areas for further development within networks to increase their effectiveness and impact.

Confirmatory factor analysis supported the seven hypothesised domains, namely: engagement of clinicians; leadership of the network manager; leadership of network co-chairs; leadership of the Agency executive; strategic and operational management of the network; external support; and value of the clinical network. The survey has high internal consistency reliability as evidenced by Cronbach’s α values of 0.75 and greater.

For this sample of nearly 600 members of 19 clinical networks of the NSW Agency for Clinical Innovation there was strong reported commitment and belief in the work that the network undertakes. Network managers were generally perceived to be effective leaders who facilitated evidence-based quality improvement initiatives and built strong working relationships with clinicians. Network co-chairs were considered to be champions for change and to have built strong, positive relationships with other clinicians. Across both manager and co-chair leadership, however, there was variability in perceived effectiveness at forming good relationships with consumers and hospital management. Further, there were perceived inconsistencies in co-chairs’ abilities to collaborate with external parties to support network operations or work cooperatively with senior health department leadership to make appropriate changes. Just over half of respondents agreed that there was strong leadership and clear strategic direction from the Agency Executive. However, more than 40 % of respondents selected a “neutral” or “don’t know” response for the two items within this domain, perhaps reflecting a lack of awareness of the higher-level operational leadership of the Agency in members with limited exposure to this level of management or members with looser affiliations to the networks.

The majority of network members were satisfied with the level of multidisciplinary representation and information sharing across the network but only a little more than half agreed that communication with people outside the network was effectively coordinated. This indicates that there may be scope for improvement in external communication to raise awareness of network initiatives and impacts. There was a perceived lack of external support for the networks, with few network members agreeing that hospital management or local area health service managers were willing to implement network recommended changes. This may be a reflection of network managers’ and co-chairs’ lesser abilities to build positive relationships and work cooperatively with these groups and could explain variation in effectiveness or success across networks. Overall, the networks were perceived as valuable and were considered by members to have improved quality of care and patient outcomes.

These results would suggest that the strength of this type of managed clinical network lies in the strategic leadership of the network manager and their ability to form constructive working relationships with clinicians working in the health system. Managers of networks seeking to improve effectiveness should seek to build stronger relationships with hospital management and local area health service managers to leverage support for network quality improvement initiatives. Given the importance of cohesion in the local community, and local community support and participation as critical factors in the success of networks [39] enhanced relationships with consumers and improved communication with those outside of the network would additionally seem important areas of focus.

It should be noted that the response rate for this Internet based survey was less than the reported average for online surveys [47]. However, respondents were split equally between participants who were recipients of network activities with a loose connection to the network (49 %), and more actively engaged members with governance or steering roles or involvement in working groups (51 %). This latter group of respondents is better placed to accurately report on the external support, organizational, and program factors measured by the survey given their greater knowledge of network functioning adding credibility to their perceptions. 55 % of respondents had been involved with the networks for five or more years suggesting a degree of commitment to the network and a proxy measure of network sustainability. While it is acknowledged that the low response rate may have impacted on the external generalisability of the instrument’s construct validity, sensitivity analyses based on inverse probability weighting to adjust for any response bias, conducted as part of the main study for which this survey was developed, [13] found correlation and regression results to be similar to the main (non-weighted) analyses.

A further potential limitation of this study is the reliance on self-reported perceptions of network members. Given the large and diverse study sample of more than 3000 members of 19 networks operating across multiple clinical areas and disciplines in a large geographical area a self-reported survey was deemed the most pragmatic, timely and cost effective method of data collection. Subjective self-reported measures were validated through document review and a sub-study, [49] and a qualitative study [50] was conducted to assist with interpretation of results.

The survey has potential for broader application beyond the context of NSW, Australia as an instrument for assessing and improving the operations of clinical networks. When other research groups outside NSW, Australia use this survey in their studies they can validate the utility and applicability of the tool and the domains selected to their contexts. Over time benchmarking and normative data across multiple jurisdictions with clinical networks could be obtained.

Given that the international literature formed the basis of the instrument, the domains measured are likely to be common across the various models of clinical networks internationally, which have the shared aims of increasing uptake of evidence-based practice and improving quality of care. A recent systematic review [51] that included both quantitative and qualitative studies of the effectiveness of clinical networks operating in other regions of Australia, Canada, the UK and other regions of Europe, and the US concluded that appropriate organisational structure, effective leadership, multidisciplinary engagement, adequate resourcing, collaborative relationships, and external support from the patient community and other stakeholders, were key features of successful clinical networks. This supports the domain structure of our instrument and suggests it’s likely generalisability beyond the current context. It should also be noted that none of the studies included in the review used a validated measure of network effectiveness, rather relying on qualitative exploration or experiential commentary, highlighting the value of this validated instrument to enable more standardised, and hence comparable, future assessment of networks.

Further, given the commonality of determinants of successful networks and core competencies for network success across different policy fields [39] there is scope for this survey to be adapted for use outside of clinical networks. For example, it could be used in the assessment of other types of public networks beyond health that deliver and manage public services such as education, job and training networks, community care or family and children’s services. The included domains relating to perceived: engagement of key stakeholders; leadership; strategic and operational management; external support; and value of the network, would all be equally applicable across these settings.

The results for this survey sample of nearly 600 network members can provide a point of comparison for others who wish to use the instrument.

## Conclusion

This survey, which has good construct validity and internal reliability, provides a valid stand-alone instrument to use in future research related to clinical networks. The survey measures seven domains of successful networks and provides managers with a means to formatively assess network functioning, identify strengths, and areas for development. Equally, the survey could be adapted for use in the context of other types of public network.

## Additional files

Additional file 1: Clinical networks survey. This file contains the clinical networks survey. (PDF 188 kb) Additional file 2: Descriptive results for the survey sample. This table provides descriptive data for the survey sample. (PDF 108 kb)

## Footnotes

1 Univariate and multivariate analyses examining the relationships between survey domains and main study outcomes, namely quality of care and system-wide change, are reported elsewhere.

## References

[1] Fleury M, Mercier C, Denis J (2002). Regional planning implementation and its impact on integration of a mental health care network. Int J Health Plann Manage.

[2] Hamilton KE, Sullivan FM, Donnan PT, Taylor R, Ikenwilo D, Scott A (2005). A managed clinical network for cardiac services: set-up, operation and impact on patient care. Int J Integr Care.

[3] Laliberte L, Fennell ML, Papandonatos G (2005). The relationship of membership in research networks to compliance with treatment guidelines for early-stage breast cancer. Med Care.

[4] McClellan WM, Frankenfield DL, Frederick PR, Flanders WD, Alfaro-Correa A, Rocco M (1999). Can dialysis therapy be improved? A report from the ESRD Core Indicators Project. Am J Kidney Dis.

[5] Ray-Coquard I, Philip T, Laroche G, Froger X, Suchaud J-P, Voloch A (2002). A controlled ‘before-after’ study: impact of a clinical guidelines programme and regional cancer network organization on medical practice. Br J Cancer.

[6] Ray-Coquard I, Philip T, Laroche G, Froger X, Suchaud J-P, Voloch A (2005). Persistence of Medical Change at Implementation of Clinical Guidelines on Medical Practice: A Controlled Study in a Cancer Network. J Clin Oncol.

[7] Spence K, Henderson-Smart D. Closing the evidence-practice gap for newborn pain using clinical networks. J Paediatr Child Health. 2011;47(3):92-8. doi:10.1111/j.1440-1754.2010.01895.x. Epub 2010 Nov 21. 10.1111/j.1440-1754.2010.01895.x 21091580

[8] Tolson D, McIntosh J, Loftus L, Cormie P (2007). Developing a managed clinical network in palliative care: a realistic evaluation. Int J Nurs Stud.

[9] Touati N, Roberge D, Denis JL, Cazale L, Pineault R, Tremblay D (2006). Clinical leaders at the forefront of change in health-care systems: advantages and issues. Lessons learned from the evaluation of the implementation of an integrated oncological services network. Health Serv Manage Res.

[10] Gale C, Santhakumaran S, Nagarajan S, Statnikov Y, Modi N, Neonatal Data Analysis U (2012). Impact of managed clinical networks on NHS specialist neonatal services in England: population based study. BMJ.

[11] Cadilhac DA, Pearce DC, Levi CR, Donnan GA (2008). Improvements in the quality of care and health outcomes with new stroke care units following implementation of a clinician-led, health system redesign programme in New South Wales, Australia. Qual Saf Health Care.

[12] Greene A, Pagliari C, Cunningham S, Donnan P, Evans J, Emslie-Smith A (2009). Do managed clinical networks improve quality of diabetes care? Evidence from a retrospective mixed methods evaluation. Qual Saf Health Care.

[13] Haines M, Brown B, Craig J, D'Este C, Elliott E, Klineberg E (2012). Determinants of successful clinical networks: the conceptual framework and study protocol. Implementation Sci.

[14] Addicott R, McGivern G, Ferlie E (2006). Networks, Organizational Learning and Knowledge Management: NHS Cancer Networks. Public Money Manage.

[15] Ahgren B, Axelsson R (2007). Determinants of integrated health care development: chains of care in Sweden. Int J Health Plann Manage.

[16] Goodwin N, 6 P, Peck E, Freeman T, Posaner R. Managing Across Diverse Networks of Care: Lessons from Other Sectors. London: NHS Service Delivery and Organisaton Programme, 2004.

[17] Hamilton KE, Sullivan FM, Donnan PT, Taylor R, Ikenwilo D, Scott A (2005). A managed clinical network for cardiac services: set-up, operation and impact on patient care. Int J Integr Care.

[18] Squires JE, Estabrooks CA, O'Rourke HM, Gustavsson P, Newburn-Cook CV, Wallin L (2011). A systematic review of the psychometric properties of self-report research utilization measures used in healthcare. Implementation Sci.

[19] American Psychological Association (1999). National Council on Measurement in Education, American Educational Research Association: Standards for educational and psychological testing.

[20] National Lead Clinicians Group established [press release]. Canberra: Australian Government, Department of Health and Ageing; 2011.

[21] Goodwin N, Perri 6, Peck E, Freeman T, Posaner R. Managing Across Diverse Networks of Care: Lessons from Other Sectors. London: National Co-ordinating Centre for NHS Service Delivery and Organisation; 2004.

[22] McInnes E, Middleton S, Gardner G, Haines M, Haertsch M, Paul CL (2012). A qualitative study of stakeholder views of the conditions for and outcomes of successful clinical networks. BMC Health Serv Res.

[23] Australian Bureau of Statistics. 3101.0 - Australian Demographic Statistics, Sep 2014. Canberra: ABS; 2015.

[24] Agency for Clinical Innovation. About ACI2013. Available from: http://www.aci.health.nsw.gov.au/. Accessed 3 Apr 2013.

[25] Braithwaite J, Goulston K (2004). Turning the health system 90° down under. Lancet.

[26] Stewart GJ, Dwyer JM, Goulston KJ (2006). The Greater Metropolitan Clinical Taskforce: an Australian model for clinician governance. Med J Aust.

[27] The Sax Institute. What have the clinical networks achieved and who has been involved? 2006-2008. Australia: Agency for Clinical Innovation; 2011. http://www.aci.health.nsw.gov.au/__data/assets/pdf_file/0018/163062/final_aci_report_280311.pdf. Accessed 3 Apr 2013.

[28] Bate P, Mendel P, Robert GB (2008). Organising for quality: the improvement journeys of leading hospitals in Europe and the United States.

[29] Braithwaite J, Westbrook JI, Ranmuthugala G, Cunningham F, Plumb J, Wiley J (2009). The development, design, testing, refinement, simulation and application of an evaluation framework for communities of practice and social-professional networks. BMC Health Serv Res.

[30] Ovretveit J (2010). Improvement leaders: what do they and should they do? A summary of a review of research. [Review]. Qual Saf Health Care.

[31] Addicott R, Ferlie E (2007). Understanding power relationships in health care networks. J Health Organ Manag.

[32] Ferlie E, Fitzgerald L, McGivern G, Dopson S, Exworthy M. Networks in Health Care: a Comparative Study of Their Management, Impact and Performance. Report for the National Institute for Health Research Service Delivery and Organisation programme. . 2010.

[33] Shipton H, Armstrong C, West M, Dawson J (2008). The impact of leadership and quality climate on hospital performance. Int J Qual Health Care.

[34] Rao PR (2006). Emotional intelligence: The Sine Qua Non for a clinical leadership toolbox. J Commun Disord.

[35] Yukl G (2002). Leadership in Organizations.

[36] Ogrinc G, Mooney SE, Estrada C, Foster T, Goldmann D, Hall LW (2008). The SQUIRE (Standards for Quality Improvement Reporting Excellence) guidelines for quality improvement reporting: explanation and elaboration. Qual Saf Health Care.

[37] Speroff T, O’Connor GT (2004). Study designs for PDSA quality improvement research. Qual Manag Health Care.

[38] Nies H, Van Linschoten P, Plaisier A, Romijn C (2003). Networks as regional structures for collaboration in integrated care for older people.

[39] Turrini A, Cristofoli D, Frosini F, Nasi G (2010). Networking literature about determinants of network effectiveness. Public Adm.

[40] Cancer Australia. Cancer service networks national demonstration program (CanNET) evaluation tool. http://canceraustralia.gov.au/clinical-best-practice/service-delivery/cannet/cannet-national-program-evaluation. Accessed 3 Apr 2013.

[41] Yano EM, Fleming B, Canelo I, et al. National Survey Results for the Chief of Staff Module of the VHA Clinical Practice Organizational Survey: Technical Monograph 07-CPOS01. In: Department of Veterans Affairs Health Services Research and Development, Center for the Study of Healthcare Provider Behavior, editor. Sepulveda, Calif, US;2007.

[42] Center for the Advancement of Collaborative Strategies in Health. Partnership self-assessment tool. United States Agency for International Development (USAID). http://www.lmgforhealth.org/node/190. Accessed 3 Apr 2013.

[43] Epley N, Gilovich T (2005). When effortful thinking influences judgmental anchoring: differential effects of forewarning and incentives on self-generated and externally provided anchors. J Behav Decis Mak.

[44] Hassan E (2006). Recall bias can be a threat to retrospective and prospective research designs. Internet J Epidemiol.

[45] Hatcher L (1994). A Step-By-Step Approach to Using the SAS System for Factor Analysis and Structural Equation Modeling.

[46] George D, Mallery P (2003). SPSS for Windows step by step: A simple guide and reference.

[47] Nulty DD (2008). The adequacy of response rates to online and paper surveys: what can be done?. Assess Eval High Educ.

[48] Bland JM (1997). Statistics notes: Cronbach’s alpha. Br Med J.

[49] Kalucy D (2013). Determinants of Effective Clinical Networks: Validation sub-study. Masters Thesis.

[50] McInnes E, Haines M, Dominello A, Kalucy D, Jammali-Blasi A, Middleton S (2015). What are the reasons for clinical network success? A qualitative study of stakeholder views. BMC Health Serv Res.

[51] Brown B, Patel C, McInnes E, Mays N, Young J, Haines M (2016). The effectiveness of clinical networks in improving quality of care and patient outcomes: A systematic review of quantitative and qualitative studies. BMC Health Serv Res.

