# Supplementary material for: Development and validation of a survey to measure features of clinical networks
Source: BMC Health Serv Res. 2016 Sep 30;16:531. doi: 10.1186/s12913-016-1800-0 (PMC5045605; doi:10.1186/s12913-016-1800-0)
Supplement: Additional file 1: — Clinical networks survey. This file contains the clinical networks survey. (PDF 188 kb) [file 12913_2016_1800_MOESM1_ESM.pdf]

NHMRC Partnership Grant
Determinants of Effective Clinical Networks

Web survey

Preamble

You have been invited to participate in this study because you have been involved with the clinical networks of the NSW Agency for Clinical Innovation (ACI) [formerly the Greater Metropolitan Clinical Taskforce (GMCT)].

The project is jointly funded by the National Health and Medical Research Council (NHMRC) and Agency for Clinical Innovation. The research is being undertaken in partnership with The Sax Institute, The Universities of Sydney, Newcastle and Melbourne and The Australian Catholic University and aims to examine the determinants of successful clinical networks.

We are interested to find out what makes some networks more successful than others. The results from this study will inform the establishment and maintenance of clinical networks so they can effectively improve the quality of care.

Participation in this survey is entirely voluntary. Submitting a completed survey is an indication of your consent to participate in the study. You can withdraw from the study at any time. All aspects of the study, including the results, will be strictly confidential. The networks will be de-identified in all reports relating to this study and will be labelled only as Networks 1-19. Network managers and chairs will not be identified by name in the publication of the results. No data will be given to ACI that identifies any network manager or chair.

If you would like further information on the study and how your responses will be used, please read the participant information sheet.

Section 1 – Linkages to ACI

From 2006 -2008

1.1 What was your primary network? (That is, the network you were most active in):

Please tick ONE:

	Network	Establishment Date	Network Manager	Co-Chairs
	Transition Care	[DATE]	[NAME]	[NAME] [NAME]
	Cardiac	[DATE]	[NAME]	[NAME] [NAME]
	Neurosurgery	[DATE]	[NAME]	[NAME] [NAME]
	Endocrine	[DATE]	[NAME]	[NAME] [NAME]
	Aged Care	[DATE]	[NAME]	[NAME] [NAME]
	Gynaecology Oncology	[DATE]	[NAME]	[NAME] [NAME]
	Radiology	[DATE]	[NAME]	[NAME] [NAME]
	Nuclear Medicine	[DATE]	[NAME]	[NAME] [NAME]
	Stroke	[DATE]	[NAME]	[NAME] [NAME]
	Home Enteral Nutrition	[DATE]	[NAME]	[NAME] [NAME]
	Urology	[DATE]	[NAME]	[NAME] [NAME]
	Gastroenterology	[DATE]	[NAME]	[NAME] [NAME]
	Ophthalmology	[DATE]	[NAME]	[NAME] [NAME]
	Respiratory	[DATE]	[NAME]	[NAME] [NAME]
	Severe Burn Injury Service	[DATE]	[NAME]	[NAME] [NAME]
	Statewide Spinal Cord Injury	[DATE]	[NAME]	[NAME] [NAME]
	Bone Marrow Transplant	[DATE]	[NAME]	[NAME] [NAME]
	Brain Injury Directorate	[DATE]	[NAME]	[NAME] [NAME]
	Renal	[DATE]	[NAME]	[NAME] [NAME]

1.2 In what year did you join the network or start to be involved? _______________________

1.3 What was your role in network?

- ☐ Chair/Executive Committee Member
- ☐ Executive & Steering Committee Member
- ☐ Expert Advisor
- ☐ Working group member
- ☐ Participant

Section 2 - Engagement

The following questions are about the importance of the network to you:

2.1 In the last 6 months how many hours have you devoted to network activities? E.g. attending network meetings, network correspondence, network quality improvement initiatives, network training activities

- ☐ <1hr
- ☐ 1-5hrs
- ☐ 5-10hrs
- ☐ 10-20hr
- ☐ 20-30hrs
- ☐ 30-40hrs
- ☐ >40hrs

2.2 On the scale provided please rate the extent to which you agree or disagree with each statement:

	strongly disagree	disagree	neither agree nor disagree	agree	strongly agree	Don't know
I am committed to the network						
I believe in the work that the network undertakes						
I am not involved in the day-to-day work of the network						
My input to the network is not highly visible but is more behind the scenes						
My views and ideas have contributed to network activities						
I have not been able to help drive the network agenda						

Section 3 - Clinical leadership

The following questions relate to the leadership of the network

3.1 Based on your personal experience, how much do you agree or disagree with each statement about the **Network Manager** **(name to be inserted)** **of your network** **(name to be inserted)**:

	strongly disagree	disagree	neither agree nor disagree	agree	strongly agree	Don't know
The network manager had an evidence-based vision						
The network manager was able to engage fellow professionals about service and quality improvement						
The network manager brought others together to facilitate action and accomplish goals						
The network manager built strong and positive relationships with clinicians						
The network manager built strong and positive relationships with consumers						
The network manager built strong and positive relationships with hospital management						
The network manager did not collaborate with external parties and administrators (e.g. AHS) to support network operations						

3.2 On the scale provided please rate the extent to which you agree or disagree with each statement about the collaborative work of the **Network Co-Chairs (names to be inserted) in your network (name to be inserted)**:

	strongly disagree	disagree	neither agree nor disagree	agree	strongly agree	Don't know
The network co-chairs did not make explicit the values and purpose of the network						
The network co-chairs were champions for change						
The network co-chairs were not able to mobilize fellow professionals about service and quality improvement						
The network co-chairs built strong and positive relationships with clinicians						
The network co-chairs built strong and positive relationships with consumers						
The network co-chairs built strong and positive relationships hospital management						
The network co-chairs did not collaborate with external parties and administrators (e.g. AHS) to support network operations						
The network co-chairs worked cooperatively with senior leadership in NSW Health to make appropriate changes						

3.3 On the scale provided please rate the extent to which you agree or disagree with each statement about the role of the former **GMCT Executive** (now known as the ACI (names to be inserted) in your network (Peter Castaldi – Chief Executive, Kate Needham – Executive Director, Carol Pollock – Chair of the Board)

	strongly disagree	disagree	neither agree nor disagree	agree	strongly agree	Don't know
The GMCT Executive provided strong leadership and clear strategic direction						
The GMCT Executive worked cooperatively with the wider health system to make appropriate changes						

Section 4 - Internal management

The following questions relate to how well you think the network was managed

On the scale provided please rate the extent to which you agree or disagree with each statement about the management of the network:

	strongly disagree	disagree	neither agree nor disagree	agree	strongly agree	Don't know
The network had multidisciplinary representation e.g. consumer, medical, nursing and allied health professionals						
The network was dominated by a few individuals						
The network provided a supportive environment allowing all voices to be heard						
The network was effective in improving information sharing across the network						
The network effectively coordinated communication with people and organizations outside the network						
The network manager had good organisational abilities						

Section 5 - Perception of external support

The following questions relate to the amount of support you believe the network received from external agencies or organisations

5.1 On the scale provided please rate the extent to which you agree or disagree with each statement about the network's relationship with **Hospital Management**:

	strongly disagree	disagree	neither agree nor disagree	agree	strongly agree	Don't know
There was strong support from hospital management for the work of my network						
Hospital management were not willing to implement changes based on the recommendations of my network						
Clinicians working in hospitals were willing to implement changes based on the recommendations of my network						

5.2 On the scale provided please rate the extent to which you agree or disagree with each statement about the networks relationship with **Area Health Services**:

	strongly disagree	disagree	neither agree nor disagree	agree	strongly agree	Don't know
AHS managers were aware of the ideas put forward by my network						
Area Health Service Managers were not willing to implement changes based on the recommendations of my network						

5.3 On the scale provided please rate the extent to which you agree or disagree with each statement about the networks relationship with **NSW Health**:

	strongly disagree	disagree	neither agree nor disagree	agree	strongly agree	Don't know
The network workplans and agendas were aligned with state government strategic plans						
NSW Health decision makers were not aware of the recommendations made by my network						

Section 6 - Perceived value

The following questions relate to how much you believe the network has made a difference:

Based on your experience, how much do you agree or disagree with each statement:

	strongly disagree	disagree	neither agree nor disagree	agree	strongly agree	Don't know
The network's efforts have improved quality of care						
The network's efforts have improved patient outcomes						
The work of the network has not led to health system improvements						
I would recommend joining this clinical network to a colleague						
The network does not help me professionally						

Section 7

We may be developing some qualitative work in the future, exploring the features of successful networks... would you be interested in being asked to participate in this work that will involve an in-depth interview?

- ☐ YES
- ☐ NO

Do you have any additional comments you would like to make?

Section 8 – About you

8.1 Gender:

- ▯ Male
- ▯ Female

8.2 What was your professional discipline (select ALL that apply):

- ▯ Medical Officer
- ▯ Nurse
- ▯ Consumer
- ▯ Allied Health
- ▯ Executive manager - non-health professional
- ▯ Researcher/academic
- ▯ Other

8.3 Where was your primary place of work? (select ONE):

- ▯ Hospital- principle referral
- ▯ Hospital – paediatric specialist
- ▯ Hospital – major non-metropolitan
- ▯ Hospital – district
- ▯ Hospital – community
- ▯ Hospital – private
- ▯ Hospital – other.
- ▯ Other (specify) ____________________________________

THANK YOU FOR YOUR TIME
