# Supplementary material for: Development and validation of a survey to measure features of clinical networks
Source: BMC Health Serv Res. 2016 Sep 30;16:531. doi: 10.1186/s12913-016-1800-0 (PMC5045605; doi:10.1186/s12913-016-1800-0)
Supplement: Additional file 2: — Descriptive results for the survey sample. This table provides descriptive data for the survey sample. (PDF 108 kb) [file 12913_2016_1800_MOESM2_ESM.pdf]

	Agree		Neutral		Disagree		Don't know	
	N	%	N	%	N	%	N	%
Perceived engagement								
I am committed to the network	344	**73.5**	83	**17.7**	33	**7.1**	8	**1.7**
I believe in the work that the network undertakes	401	**85.7**	40	**8.6**	19	**4.1**	7	**1.5**
I am not involved in the day-to-day work of the network	356	**76.1**	54	**11.5**	52	**11.1**	6	**1.3**
My input to the network is not highly visible but is more behind the scenes	241	**51.5**	119	**25.4**	82	**17.5**	25	**5.4**
My views and ideas have contributed to network activities	258	**55.2**	107	**22.9**	77	**16.5**	25	**5.4**
I have not been able to help drive the network agenda	145	**31.0**	134	**28.6**	169	**36.1**	20	**4.3**
Perceived leadership of network manager								
The network manager had an evidence-based vision	303	**71.0**	58	**13.6**	17	**4.0**	49	**11.5**
The network manager was able to engage fellow professionals about service and quality improvement	314	**73.5**	43	**10.1**	28	**6.6**	42	**9.8**
The network manager brought others together to facilitate action and accomplish goals	324	**75.9**	41	**9.6**	24	**5.6**	38	**8.9**
The network manager built strong and positive relationships with clinicians	305	**71.4**	56	**13.1**	25	**5.9**	41	**9.6**
The network manager built strong and positive relationships with consumers	209	**49.1**	104	**24.4**	20	**4.7**	93	**21.8**
The network manager built strong and positive relationships with hospital management								
The network manager did not collaborate with external parties and administrators (e.g. AHS) to support network operations	44	**10.3**	82	**19.2**	186	**43.6**	115	**26.9**

	Agree		Neutral		Disagree		Don't know	
	N	**%**	N	**%**	N	**%**	N	**%**
Perceived leadership of network co-chairs								
The network co-chairs did not make explicit the values and purpose of the network	52	**12.8**	81	**20.0**	209	**51.5**	64	**15.8**
The network co-chairs were champions for change	259	**63.8**	68	**16.8**	25	**6.2**	54	**13.3**
The network co-chairs were not able to mobilize fellow professionals about service and quality improvement	59	**14.5**	84	**20.7**	194	**47.8**	69	**17.0**
The network co-chairs built strong and positive relationships with other clinicians	250	**61.6**	71	**17.5**	22	**5.4**	63	**15.5**
The network co-chairs built strong and positive relationships with consumers	161	**39.7**	109	**26.9**	22	**5.4**	114	**28.1**
The network co-chairs built strong and positive relationships hospital management	128	**31.5**	114	**28.1**	33	**8.1**	131	**32.3**
The network co-chairs did not collaborate with external parties and administrators (e.g. AHS) to support network operations	26	**6.4**	81	**20.0**	171	**42.1**	128	**31.5**
The network co-chairs worked cooperatively with senior leadership in NSW Health to make appropriate changes	210	**51.7**	73	**18.0**	19	**4.7**	104	**25.6**
Perceived leadership of the Agency Executive								
The GMCT Executive provided strong leadership and clear strategic direction	217	**53.8**	92	**22.8**	17	**4.2**	77	**19.1**
The GMCT Executive worked cooperatively with leaders in the wider health system to make appropriate changes	223	**55.3**	84	**20.8**	16	**4.0**	80	**19.9**

	Agree		Neutral		Disagree		Don't know	
	N	**%**	N	**%**	N	**%**	N	**%**
Perceived strategic and operational management								
The network had multidisciplinary representation e.g. consumer, medical, nursing and allied health professionals	318	**81.8**	29	**7.5**	15	**3.9**	27	**6.9**
The network was dominated by a few individuals	107	**27.5**	110	**28.3**	138	**35.5**	34	**8.7**
The network provided a supportive environment allowing all voices to be heard	249	**64.0**	78	**20.0**	31	**7.0**	31	**8.0**
The network was effective in improving information sharing across the network	292	**75.1**	56	**14.4**	20	**5.1**	21	**5.4**
The network effectively coordinated communication with people and organisations outside the network	217	**55.8**	77	**19.8**	33	**8.5**	62	**15.9**
The network manager had good organisational abilities	281	**72.2**	52	**13.4**	16	**4.1**	40	**10.3**
Perceived external support								
There was strong support from hospital management for the work of my network	108	**28.6**	112	**29.6**	85	**22.5**	73	**19.3**
Hospital management were not willing to implement changes based on the recommendations of my network	70	**18.5**	133	**35.2**	95	**25.1**	80	**21.1**
Clinicians working in hospitals were willing to implement changes based on the recommendations of my network	190	**50.3**	95	**25.1**	30	**7.9**	63	**16.7**
AHS managers were aware of the ideas put forward by my network	130	**34.4**	76	**20.1**	54	**14.3**	118	**31.2**
AHS managers were not willing to implement changes based on the recommendations of my network	62	**16.4**	135	**35.7**	60	**15.9**	121	**32.0**

	Agree		Neutral		Disagree		Don't know	
	N	**%**	N	**%**	N	**%**	N	**%**
The network workplans and agendas were aligned with state government strategic plans	180	**52.5**	60	**17.5**	18	**5.2**	85	**24.8**
NSW Health decision makers were not aware of the recommendations made by my network	34	**9**	85	**22.5**	134	**35.5**	125	**33.1**
Network perceived as valuable								
The networks efforts have improved quality of care	273	**72.8**	50	**13.3**	23	**6.1**	29	**7.7**
The networks efforts have improved patient outcomes	237	**63.2**	69	**18.4**	25	**6.7**	44	**11.7**
The work of the network has not led to health system improvements	61	**16.3**	63	**16.8**	215	**57.3**	36	**9.6**
I would recommend joining this clinical network to a colleague	269	**71.7**	68	**18.1**	29	**7.7**	9	**2.4**
The network does not help me professionally	65	**17.4**	75	**20.1**	228	**61.0**	6	**1.**
